# Isolation of Monoclonal Antibodies with Predetermined Conformational Epitope Specificity

**DOI:** 10.1371/journal.pone.0038943

**Published:** 2012-06-21

**Authors:** Anton M. Sholukh, Muhammad M. Mukhtar, Michael Humbert, Sosthène S. Essono, Jennifer D. Watkins, Hemant K. Vyas, Vivekanandan Shanmuganathan, Girish Hemashettar, Maria Kahn, Shiu-Lok Hu, David C. Montefiori, Victoria R. Polonis, Peter H. Schur, Ruth M. Ruprecht

**Affiliations:** 1 Department of Cancer Immunology and AIDS, Dana-Farber Cancer Institute, Boston, Massachusetts, United States of America; 2 Harvard Medical School, Boston, Massachusetts, United States of America; 3 Department of Pharmaceutics, University of Washington, Seattle, Washington, United States of America; 4 Department of Microbiology, University of Washington, Seattle, Washington, United States of America; 5 Department of Surgery, Duke University School of Medicine, Durham, North Carolina, United States of America; 6 The Military HIV Research Program, Walter Reed Army Institute of Research, Silver Spring, Maryland, United States of America; 7 Department of Rheumatology, Immunology and Allergy, Brigham and Women’s Hospital, Boston Massachusetts, United States of America; University of Massachusetts Medical Center, United States of America

## Abstract

Existing technologies allow isolating antigen-specific monoclonal antibodies (mAbs) from B cells. We devised a direct approach to isolate mAbs with predetermined conformational epitope specificity, using epitope mimetics (mimotopes) that reflect the three-dimensional structure of given antigen subdomains. We performed differential biopanning using bacteriophages encoding random peptide libraries and polyclonal antibodies (Abs) that had been affinity-purified with either native or denatured antigen. This strategy yielded conformational mimotopes. We then generated mimotope-fluorescent protein fusions, which were used as baits to isolate single memory B cells from rhesus monkeys (RMs). To amplify RM immunoglobulin variable regions, we developed RM-specific PCR primers and generated chimeric simian-human mAbs with predicted epitope specificity. We established proof-of-concept of our strategy by isolating mAbs targeting the conformational V3 loop crown of HIV Env; the new mAbs cross-neutralized viruses of different clades. The novel technology allows isolating mAbs from RMs or other hosts given experimental immunogens or infectious agents.

## Introduction

Isolation of naturally induced and matured antibodies (Abs) is of prime importance for analytical vaccinology [1], [2]. Three major strategies have been used to interrogate the B-cell repertoire: traditional phage display, high-throughput screening of immortalized B cell or plasma cell cultures, and isolation of antigen-specific B cells to PCR amplify the variable heavy (V_H_) and variable light (V_L_) immunoglobulin (Ig) genes [2], [3]. Development and refinement of high-throughput screening methods, flow cytometric capabilities and single-cell cloning techniques led to substitution of the traditional phage display techniques by approaches that allow the isolation of naturally selected Igs. Phage display is constrained by the diversity of the library used, by physical-chemical properties of the Ig fragments displayed and involves random combination of V_H_/V_L_ pairs. Consequently, it is not known whether Abs isolated by phage display represent natural molecules generated by the host in response to immunization or infection by a pathogen of interest. Even the recently published technique based upon high-throughput DNA sequencing of the plasma cell Ig repertoire with subsequent comprehensive analysis of V_H_ and V_L_ representation resulting in the assembly of V_H_/V_L_ pairs left the possibility that the recovered pair may be artificial [4].

Naturally occurring Abs can be isolated via high-throughput screening of immortalized memory B or plasma cell cultures. This approach yielded broad and potent neutralizing monoclonal Abs (nmAbs) against HIV, dengue and influenza viruses that significantly improved our understanding of the specificity and mechanisms of humoral immunity [5]–[8]. Isolation of antigen-specific B cells by sorting with a fluorescence-activated flow cytometer (FACSorting), first reported in 1972 [9], has led to the recovery of a number of naturally induced and matured Abs against rotavirus and HIV [10]–[12]. However, using the whole antigen for B-cell sorting implies a need for extensive screening of newly isolated mAbs, followed by epitope mapping. A more sophisticated approach consists of directly isolating B cells specific for individual epitopes. The success of this approach depends on the identification of epitope mimetics (mimotopes) that correctly reflect the three-dimensional structure of a given antigen subdomain/epitope. Here we report the development of a novel methodology using fluorescently labeled mimotopes as baits to isolate rhesus monkey (RM) memory B cells for subsequent cloning of Ab genes.

## Results

### Overview of Strategy

**Figure 1:**
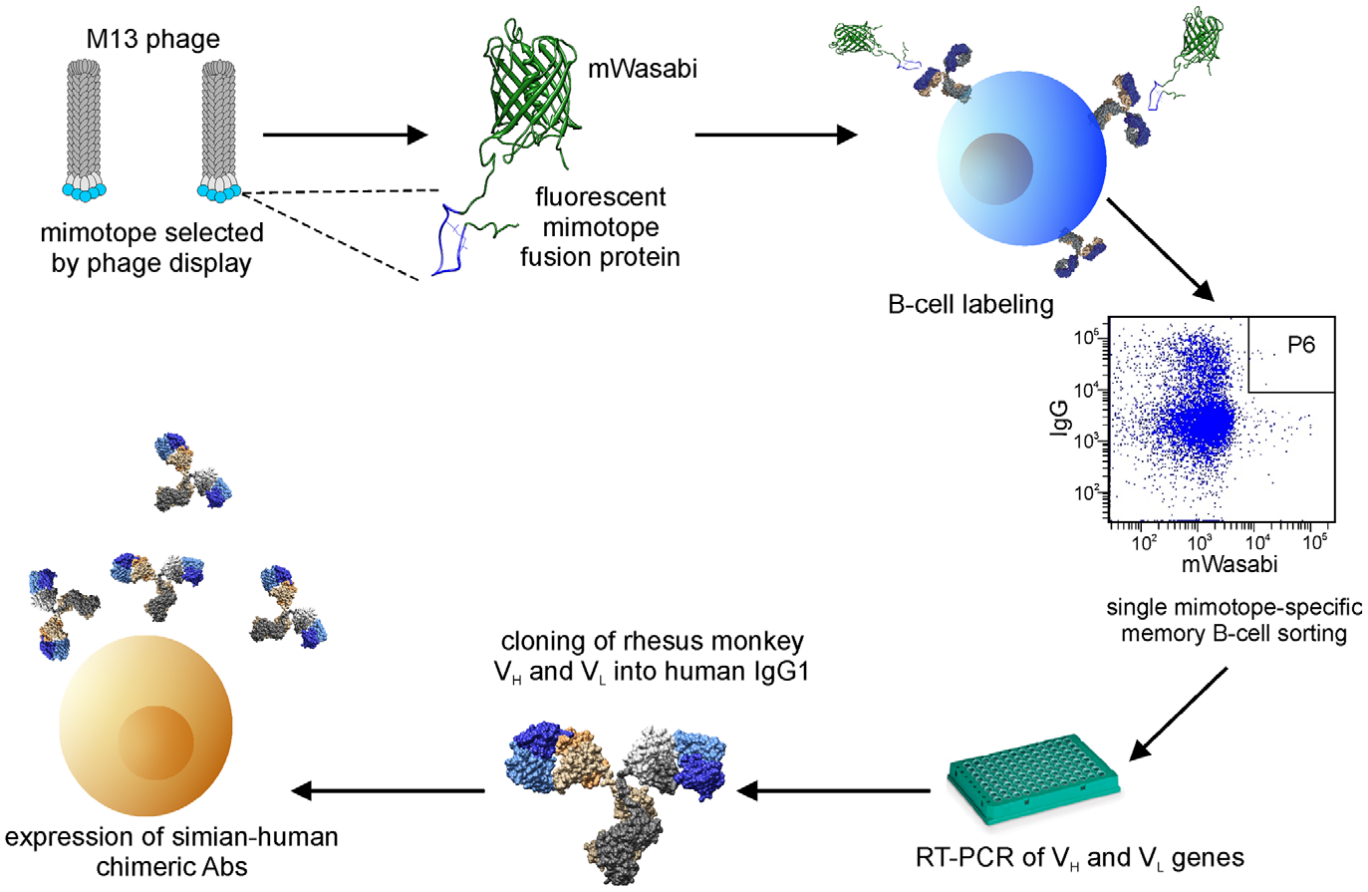
Strategy of isolation of epitope-specific Abs. After differential biopanning of phage display peptide library, mimotopes representing conformational epitopes are sequenced, and the inserts plus M13 flanking sequences are cloned into a bacterial expression vector encoding fluorescent protein. After purification of the resulting mimotope fusion proteins, binding assays to assure maintenance of the correct three-dimensional structure of mimotope insert are conducted. This is followed by single-cell sorting for specific IgG-positive memory B cells, single-cell RT-PCR and cloning of RM V_L_ and V_H_ regions into pFUSE2-type vectors encoding the backbone of human IgG1.

**Figure 2:**
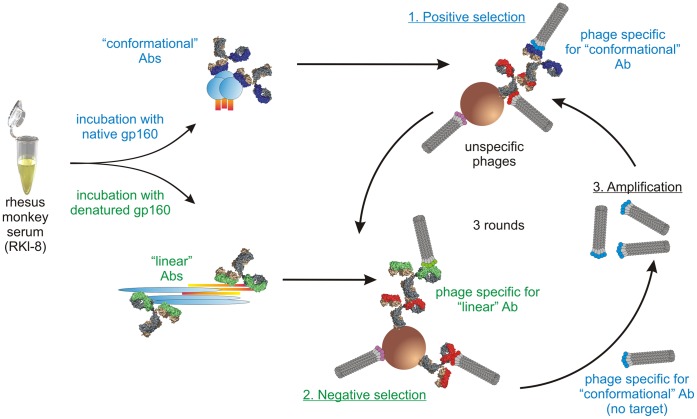
Differential biopanning strategy to select conformational mimotopes of desired specificity. HIV gp160 was bound to ELISA plates under native or denaturing conditions. After incubation with serum of monkey RKl-8 (which was chronically infected with a clade C simian-human immunodeficiency virus (SHIV) [25] and had high titers of anti-HIV nAbs) followed by washing, Abs were eluted. Abs eluted from the native protein antigen (“conformational” Abs) were used for the positive selection rounds; Abs eluted from the denatured protein (“linear” Abs) were used for the negative rounds. A total of three biopanning rounds were performed and mimotopes selected by this strategy were tested for specificity and conformation dependence with rhesus monkey sera.

The overall strategy is depicted in Figure 1. Mimotopes were isolated by biopanning using recombinant bacteriophages encoding random peptide libraries. To enrich for conformational mimotopes, we devised a new strategy of differential biopanning that involved positive selection with Abs pre-purified on native antigen followed by negative selection with Abs eluted from denatured antigen (Figure 2). After a total of three rounds of positive/negative selections with these Ab preparations, the resulting mimotopes are expected to show preferential conformation dependence. Next, mimotope sequences are cloned into a vector encoding a fluorescent reporter gene, giving rise to a fluorescent fusion protein (Figure 1). The latter is used for mimotope-specific isolation of single memory B cells by flow cytometry. Single-cell RT-PCR is then used to amplify the V_H_ and V_L_ Ig genes using newly designed, RM-specific primers. Next, the V_H_ and V_L_ sequences are cloned into expression vectors encoding the backbone of human IgG1 heavy or light chains, respectively. Cotransfection into eukaryotic cells grown in serum-free medium, will yield recombinant, chimeric simian-human monoclonal Abs (mAbs). Such mAbs will specifically recognize not only the fluorescently labeled mimotope fusion protein used for the selection procedure, but also the corresponding antigen, with the epitope specificity determined by the nature of the mimotope used for the selection.

### Isolation of Conformational Mimotopes

To validate our new strategy, we sought to isolate mAbs specific for the V3 loop of the HIV envelope glycoprotein, using conformation-dependent mimotopes. The V3 loop is a prime target for nAbs and has conserved structural features in the crown, although the entire loop structure is known for its remarkable sequence variability [13]. Anti-V3 mAbs isolated and characterized by others [14]–[17] allowed us to compare and validate the technology presented here.

**Figure 3:**
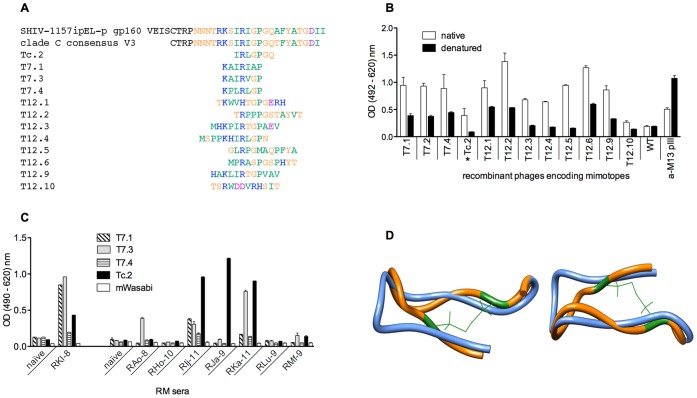
Properties of V3 loop mimotopes. (A) Binding of rhesus monkey RKl-8 serum Abs to native and denatured mimotopes. Wild-type phages (WT) were used as negative control, an anti-phage Ab (anti-M13 pIII) as positive control to show correct phage immobilization. Each data point represents the mean ± s.e.m. (n  = 3). *Mimotope selected for B-cell sorting. (B) Sequence alignment of V3 loop region of gp160 and mimotopes isolated by differential biopanning. Amino acid colors indicate: blue, basic; green, non-polar (hydrophobic); orange, polar (uncharged); magenta (acidic). (C) Binding of RM sera to different V3 mimotopes. Plates were coated with mWasabi-mimotope fusion proteins and parental mWasabi as control and then probed with sera of rhesus monkeys with high nAb titers. Serum from monkey RKl-8 served as positive control; serum from a naïve animal was used as negative control in all experiments. Each data point represents the mean ± s.e.m. (n  = 3). (D) Structural superimposition of Tc.2 mimotope and V3 peptide obtained by *in silico* molecular modeling. Tc.2 mimotope was modeled using the published V3 peptide structure in complex with Fab2557 [13]. The original V3 loop is shown in blue, the Tc.2 mimotope in orange. The cysteine residues and disulfide bridge are in green.

We used RMs that had developed high-titer, broadly neutralizing Abs (nAbs) during chronic infection with simian-human immunodeficiency virus (SHIV) (Table S1 and Table S2) for B-cell isolation. First, we affinity-purified polyclonal Abs that contained paratopes specific for conformational epitopes on the original antigen molecule. In parallel, we also affinity-purified polyclonal Abs recognizing the same antigen in denatured form. Towards this end (Figure 2), wells of 96-well plates were coated with HIV gp160 under native or denaturing conditions and incubated with serum of monkey RKl-8. Bound Abs were eluted by pH shift, identical fractions were combined, concentrated and used for biopanning of random peptide phage libraries. “Conformational” Abs (eluted from native, multimeric gp160, shown in blue, Figure 2) were used for positive selection; “linear” Abs (eluted from denatured gp160, shown in green, Figure 2) for negative selection. Three positive/two negative rounds yielded mimotopes that were tested for gp160 specificity and conformational dependence with RM sera. Interestingly, only 12 mimotopes resembling the V3 loop crown of HIV gp120 (Figure 3A) were selected using this method of differential biopanning with Abs specific for either native or denatured gp160. Some of these conformational V3 mimotopes were chosen to test the feasibility of our overall strategy. Notably, our differential biopanning strategy resulted in the selection of mimotopes with strong conformational dependence as shown by ELISA performed with native or denatured phages and probed with serum from RM RKl-8 (Figure 3B). The fact that only V3-like mimotopes were found may be ascribed to the stringent selection conditions. It should be noted that mimotopes positively selected by the “conformational” Abs (shown in blue, Figure 2) are discarded in the subsequent negative selection step if they are also bound to “linear” Abs (shown in green, Figure 2). As a consequence, the only mimotopes that remain are those with high specificity and selective binding to the “conformational” Abs, a condition fulfilled by the sterically constrained V3 loop.

Next, we cloned, bacterially expressed and purified mWasabi-mimotope fusion proteins (termed mWasabi-mimes, Figure S1). We analyzed the latter by ELISA with sera from animals with high-titer nAbs to confirm specificity and cross-recognition. Mimotope Tc.2 was recognized by serum of different RMs and serum of monkey RJa-9 demonstrated the highest binding (Figure 3C). This mimotope closely resembles the V3 loop crown in amino acid sequence (Figure 3A). Conformational similarity of mimotope and V3 loop is demonstrated by molecular modeling and structural superimposition shown on Figure 3D. Noteworthy, Tc.2 was isolated from cyclic 7mer peptide library and is flanked by two cysteines forming disulfide bridge thus stabilizing the mimotope structure.

### Isolation of Single Memory B Cells

**Figure 4:**
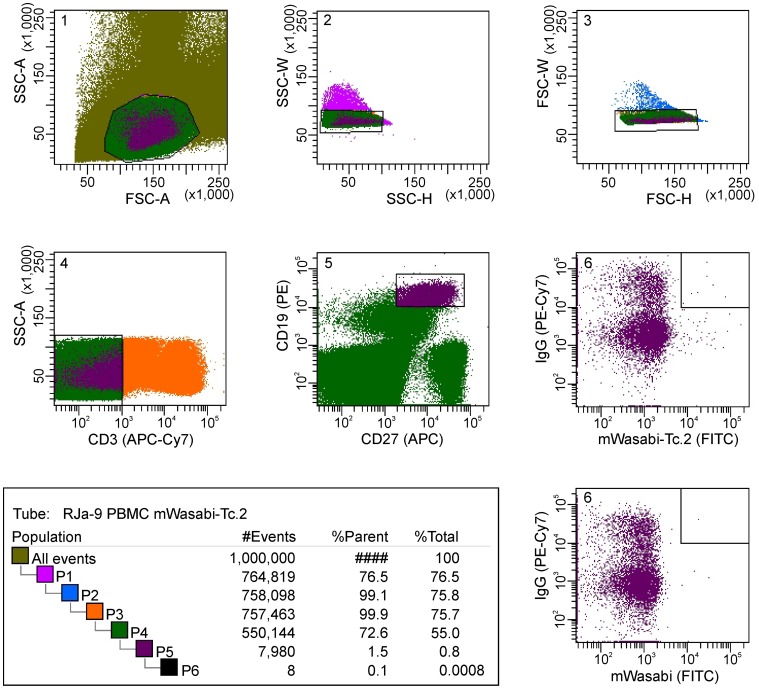
Isolation of single V3 loop-specific memory B cells by FACSorting. Mimotope-specific memory B cells were selected by the following phenotype: CD3^−^/CD19^+^/CD27^+^/IgG^+^/mWasabi-mimotope^+^ and were sorted into individual wells of a 96-well PCR plate. The consecutive number of gates is shown in the left upper corner of each gate.

The cross-reactivity of mimotope Tc.2 with sera from different RMs implied mimicry of a conserved HIV Env epitope by Tc.2. Consequently, we selected the corresponding mWasabi-Tc.2 fusion protein as bait to isolate cognate memory B cells from PBMC of monkey RJa-9 by flow cytometry. Mimotope-specific single memory B cells were sorted into a PCR plate as follows: CD3^−^/CD19^+^/CD27^+^/IgG^+^/mWasabi-mime^+^ (Figure 4). For the experiment 5×10^7^ PBMC were used and the cytometer was programmed to collect 90 individual CD3^−^/CD19^+^/CD27^+^/IgG^+^/mWasabi-mime^+^ cells at one cell per well in the plate. Mimotope-specific cells represented approximately 0.1% of the memory B cells and as many as 8 mimotope-specific IgG-positive memory B cells were observed per million lymphocytes sorted. Control staining with mWasabi yielded only one non-specific cell per million lymphocytes, possibly due to autofluorescence.

### Single-cell PCR and mAb Cloning

After cDNA synthesis, light and heavy chain variable Ig genes were amplified by semi-nested PCR with a set of newly developed primers specific to RM Ig genes (Table S3). RM IgG-specific primers published to date do not result in the amplification of V_H_ and V_L_ genes of all RM subfamilies [18], whereas our new primer set led to the amplification of all RM V_H_ and V_L_ genes and yielded frequencies similar to their natural usage rate (Table S4). This procedure allowed us to derive V_H_ gene fragments in 31 wells and V_L_ gene fragments in 19 wells, respectively. In 12 wells, both V_H_ and V_L_ genes were amplified.

Next, we sequenced matching pairs of V_H_ and V_L_ genes obtained after two rounds of PCR to assess productivity and gene rearrangement as well as to obtain sequence information for the beginning of framework region 1 (FR1). After amplification of V_H_/V_L_ pairs with cloning primers, we inserted the PCR fragments into vectors of the pFUSE2-family that contain constant region sequences of human Ig light (Igκ or Igλ2) or heavy (Igγ1) chains. This cloning strategy yielded chimeric simian-human IgG1 mAbs.

### Functional Characterization of mAbs

**Figure 5:**
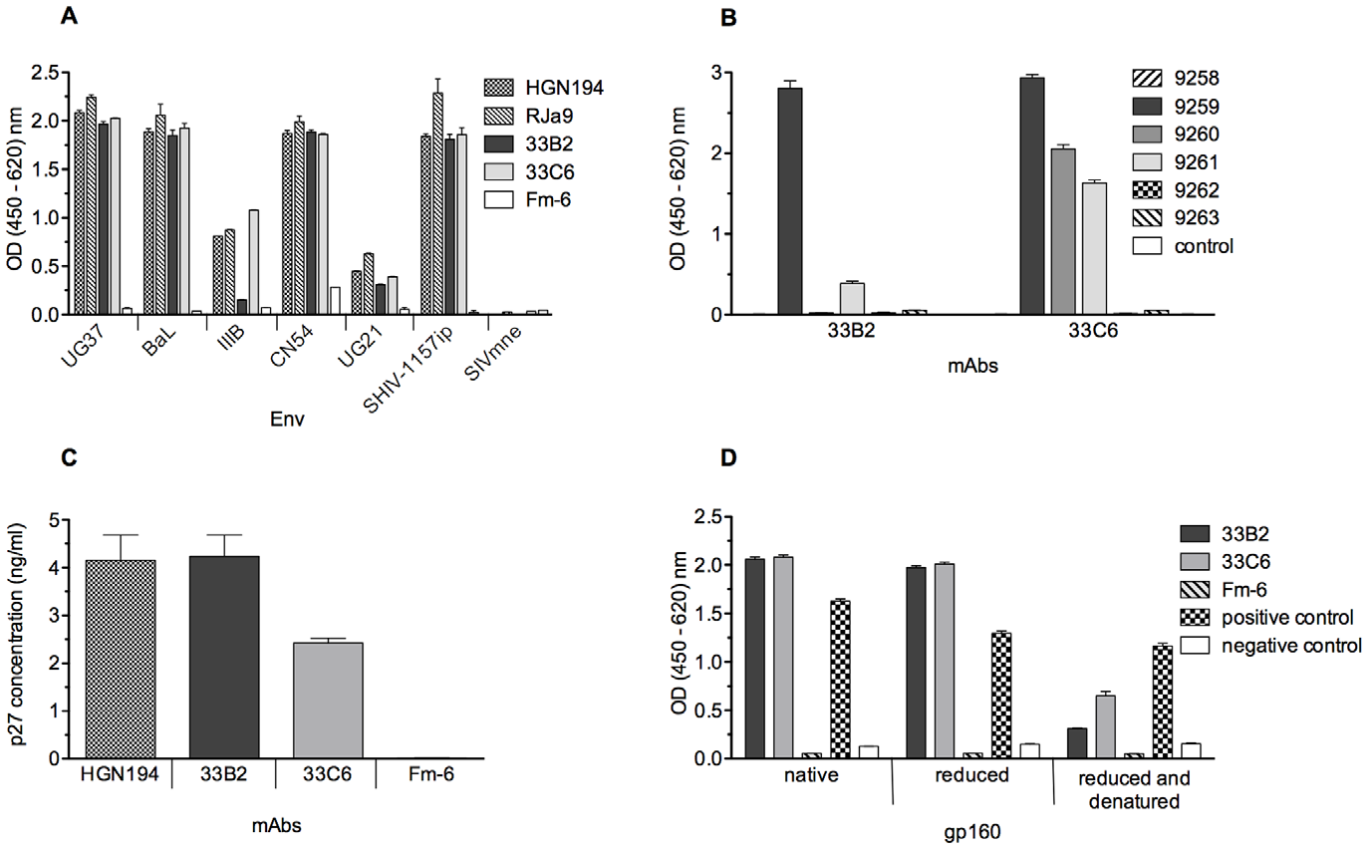
Properties of mAbs 33B2 and 33C6. (A) Binding to envelope proteins of different HIV clades. Plates were coated with Env proteins and probed with newly isolated mAbs along with isotype controls (positive control, HGN194 [16]; negative control, Fm-6 [37]) and cognate rhesus monkey (RJa-9) serum. HIV Env proteins were derived from the following strains: clade A, UG37; B, BaL and IIIB; C, CN54 and 1157ip (the latter is a simian-human immunodeficiency virus (SHIV) strain encoding an HIV clade C envelope [25]; D, UG21. SIVmne gp160 was used as negative control. (B) Epitope mapping. Bars show Ab binding by ELISA to consensus HIV clade C peptides representing the V3 loop region. The control peptide represents the scrambled C-terminal sequence of SHIV-1157ip gp120. (C) Virion binding assay. MAbs 33B2 and 33C6 along with negative control (Fm-6) and positive control (HGN194) were captured on the plate coated with goat anti-human IgG Fc-specific Ab and then exposed to SHIV-1157ipEL-p [19] virions. The amount of virus bound to mAbs was assessed by p27 Gag ELISA. (D) Binding of mAbs 33B2 and 33C6 with native and denatured HIV_CN54_ gp120. Plates were coated with native, reduced or reduced/denatured HIV Env protein and probed with 33B2 and 33C6 along with Fm-6 (negative control mAb) as well as serum of HIV-positive individual (positive control), and serum of HIV-negative individual (negative control). We used *tris*(2-carboxyethyl)phosphine (TCEP) to reduce gp120 and TCEP + SDS to reduce and denature, respectively (Methods). Each data point represents the mean ± s.e.m. (n  = 3). Experiments were repeated 3 times.

Out of 12 isolated V_H_/V_L_ pairs, 11 chimeric mAbs were expressed and purified. Two of them, 33B2 and 33C6, exhibited specific binding to the Tc.2 mimotope and HIV-1_CN54_ gp120 (Figure S2) as well as HIV envelopes of different clades by ELISA (Figure 5A, Figure S3). The weaker binding of mAbs to Tc.2 compare to gp120 (Figure S2), especially 33B2, can be explained by the fact that the mimotope represents only part of entire epitope due to the relatively short length.

To confirm the expected V3 loop epitope specificity of mAbs 33B2 and 33C6, peptides representing the V3 loop of consensus HIV clade C gp120 were used for epitope mapping. Peptide binding analysis revealed a common epitope recognized by both mAbs (Figure 5B, Figure S4A): the sequence RKSIRIG, which is located in the V3 loop crown and resembles the epitope of the known anti-V3 mAb, HGN194 [16]. Interestingly, mAb 33B2 interacted only with peptides 9259 and 9261 bearing the epitope either at the N- or C-termini but did not bind to peptide 9260, where the epitope is in the middle, presumably due to improper conformation of the linear peptide. Peptide 9261 showed the highest degree of inhibition of mAbs binding to gp120 (Figure S4B and S4C). Lower binding of mAb 33B2 compared to binding of 33C6 to linear peptides 9260 and 9261 implies that epitope of 33B2 is more conformation-dependent.

Since the mimotope used as B-cell bait was selected to be strongly conformational, we postulated that 33B2 and 33C6 would preferentially bind to native as opposed to denatured envelope proteins. To confirm binding to native Env in trimeric form, we performed a virion binding assay, in which both mAbs successfully captured virions of the chimeric simian-human immunodeficiency virus (SHIV) encoding *env* of a recently transmitted HIV clade C, SHIV-1157ipEL-p [19] (Figure 5C). Upon Env denaturation, binding of both mAbs was strongly diminished by ELISA (Figure 5D); denaturation greatly abrogated binding of both mAbs to gp120, whereas reduction of disulfide bridges did not affect binding.

Next, we assessed potency/breadth of virus neutralization of the two mAbs with a panel of neutralization-sensitive (tier 1) and more difficult-to-neutralize (tier 2) viruses. Overall, 33B2 and 33C6 neutralized 75% or 91% of the tier 1 HIV strains of different clades tested (Table 1), respectively. In contrast, few of the tier 2 strains tested were neutralized by the two new mAbs (7% and 15%, respectively). These results are similar to the neutralization profiles of other anti-V3 Abs [15]–[17]. The fact that we preselected conformation-dependent mimotopes increased our chances of finding neutralizing mAbs since most virus-neutralizing Abs are conformation dependent.

**Table 1:** Neutralization activity of mAbs 33B2 and 33C6.

			33B2	33C6
Virus	Clade	Tier	IC_50_ (µg/ml)	IC_50_ (µg/ml)
*SHIV strains (M7-Luc assay)*				
SHIV-KNH1144p	A	2	>20	>20
SHIV_SF162.P4_	B	1	3.98	0.02
SHIV-1157ipEL-p	C	1	0.05	0.06
SHIV-1157ipd3N4	C	2	ND	>20
SHIV-2873Nip	C	2	>20	>20
*HIV strains (TZM-bl assay)*				
Q23.17	A	1	>20	>25
Q259.d2.17	A	2	>20	>25
Q769.d22	A	2	>20	>25
92UG029	A/X4	1/2	ND	0.02
SF162.LS	B	1	0.3	0.05
BaL	B	1	>20*	0.06*
MN.3	B	1	0.02	<0.01
BX08	B	1	ND	0.76
6535.3	B	2	>20	>25
RHPA	B	2	(>20)	(1.2)
SC22.3C2	B	2	(>20)	(>25)
QH0692.42	B	2	>20	>25
MW965.26	C	1	<0.01	<0.01
92BR025.9	C	1	0.9	0.6
GS015	C	1	ND	0.05
Ce1086_B2	C	2	(17.9)	(>25)
Du151.2	C	2	(>20)	(>25)
Du156.12	C	2	>20	>25
CAP45.2.00.G3	C	2	>20	>25
Indie-C1	C	2	>20*	>25*
20635-4	C	2	ND	13.0
E0836M4	D	2	ND	>50
R2184.c04	AE	2	(>20)	(>25)
CM235-2	AE	2	(>20)	(>25)
CM235	AE	2	ND	27.26
55815	AG	2	ND	>50
overall tier 1 neutralization			75%	91%
overall tier 2 neutralization			7%	15%

Neutralization assays were performed in M7-Luc cells (for SHIVs) or TZM-bl cells (for HIVs) [33] unless otherwise mentioned; values in brackets show neutralization assays with A3R5.7 cells [36]. *Indicates HIV neutralization in M7-Luc assay. SHIV, simian-human immunodeficiency virus. ND, not determined.

### Genetic Analysis of mAbs

**Figure 6:**
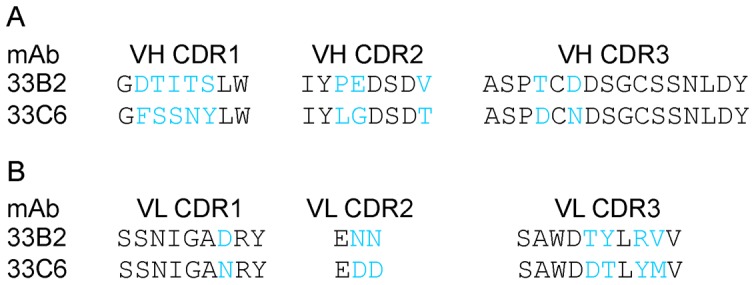
VH and VL CDR sequences of V3-specific mAbs. (A) 33B2 and 33C6 VH CDR amino acid sequences. (B) 33B2 and 33C6 VL CDR amino acid sequences.

Genetic analysis of 33B2 and 33C6 V_H_ and V_L_ genes revealed that these mAbs likely originated from the same parental B cell clone, because they share the same V_H_ and J_H_ genes, IGHV5-51*01 and IGHJ4*2, respectively (Table 2). Although 33B2 and 33C6 used different D genes, the complementarity-determining region 3 (CDR3) of both mAbs are 16 amino acids long and have very similar amino acid sequences (Figure 6). In contrast, the light chains recruited different genes, IGLV1-50 and IGLV1-47, from the same subfamily (Table 2). The CDR sequence homology indicates convergence of the molecular evolution of both mAbs (Figure 6). Our results are in good agreement with data about Ig gene usage by other anti-V3 Abs [14]. Compared to the RM germline, the frequency of amino acid mutations observed in VH was 15% for mAb 33B2 and 18% for 33C6, whereas for VL, the mutation frequencies were 10% and 15%, respectively (Figure S5 and Figure S6). These values are significantly lower compared to the recently identified anti-CD4 binding site neutralizing mAbs, but comparable to the parameters of other anti-V3 mAbs and of mAb b12 [11], [12], [16], [20]–[23]. Neither mAb exhibited autoreactivity in assays involving double-stranded (ds) DNA, Sm, ribonucleoprotein (RNP), SS-A/Ro and SS-B/La antigens, cardiolipin and nuclear antigens (data not shown).

**Table 2:** Genetic characteristics of V3-specific mAbs.

	Heavy chain genes			Light chain genes		
mAb	V_H_	D_H_	J_H_	V_κ_/V_λ_	J_κ_/J_λ_	κ/λ
33B2	IGHV5-51*01	IGHD3-22*01	IGHJ4*02	IGLV1-50*01	IGLJ3*01	λ
33C6	IGHV5-51*01	IGHD5-12*01	IGHJ4*02	IGLV1-47*02	IGLJ3*02	λ

## Discussion

Our data give proof-of-concept that the novel mAb isolation strategy, together with the newly developed set of primers specific to RM Ig genes, yielded mAbs with the epitope specificity as predicted by the fluorescently labeled mimotope. Our methodology represents a direct approach to isolate naturally occurring, matured Abs as mAbs without the need of extensive screening and provides a practical tool for analytical vaccinology. In contrast to other methods of mAb generation using phage display with Ig fragments that yield randomly recombined Ig chains, isolating single memory B cells will allow probing of natural Ab repertoires. Our focus on isolating peripheral blood memory B cells as preferred source of matured V_H_/V_L_ genes has an advantage compared to plasma cells. The latter lack B-cell receptors (BCR), whereas memory B cells bear antigen receptors recognizing only one epitope; these receptors serve as targets that are specifically recognized by fluorescently labeled mimotopes.

An important aspect of our new technology is the use of conformational mimotopes, which we selected with affinity-purified polyclonal RM Abs specific for either native or denatured antigen. We used the power of differential biopanning to select mimotopes representing conformational epitopes and thus increased the chances of isolating biologically relevant V_H_/V_L_ genes, since most Ab paratopes are directed against conformational targets on the antigen.

Our new approach to isolate conformation-specific mAbs with predetermined epitope specificity is applicable to any antigen. General strategies depicted in Figures 1 and 2 are not restricted to isolating HIV envelope-related mAbs. We chose a V3 mimotope to illustrate the power and practicality of our new approach. By opting to isolate mAbs with a narrow, well defined epitope specificity, we expect to observe far fewer “hits” when isolating memory B cells compared to standard technologies using entire antigens as baits. The benefits of our targeted approach will be that the epitope specificity will be known, thereby obviating a labor-intensive mapping effort. Another approach to zero in on a specific antigen region involved an HIV gp120 that had been redesigned to diminish undesirable antigenic determinants to facilitate the isolation of B cells specific for the CD4-binding site [12]. The latter is known to encompass a number of different epitopes. This approach required resolving of spatial structure of the antigen as well as sophisticated protein modeling. In contrast, empirical selection of epitope mimetics by differential biopanning of phage display peptide libraries using polyclonal Abs provides a fast and easy technique to generate mimotopes resembling conformational epitopes from infected or immunized hosts.

It should be pointed out that our single cell-based mAb isolation does not depend on using mimotopes with stringent conformation dependence as baits. In fact, we have performed a number of RM B-cell isolations with mimotopes that had some but not exclusive conformation dependence (unpublished data). In fact, we have performed biopannings with more than 20 RM sera from vaccinated and/or SHIV-infected animals using standard rather than differential biopanning. In such cases, we are generally able to assign the resulting mimotopes to the corresponding entire antigen of interest in approximately 90% of the cases based upon partial amino acid similarity. However, in exceptional cases, as many as 40% to 50% of the mimotopes could not be assigned and are likely representing quaternary conformational regions.

Our choice of RMs as source for V_H_/V_L_ genes will potentially yield mAbs with clinical applicability as prophylactic or therapeutic agents. Of note, RMs and humans have a closely related repertoire of Ig genes, much more so than mice, rabbits or other species used routinely for mAb isolation. Our findings of V_H_ gene usage, length of CDR3 and frequency of mutations are similar to anti-V3 Abs derived from HIV-infected individuals, thus confirming this notion.

Although we demonstrated the ability to generate mAbs with predetermined epitope specificity using the RM model of immunodeficiency virus infection, our approach can be readily extended to any target of choice, such as experimental or natural infection with potential pathogens, autoimmune diseases and cancer.

## Materials and Methods

### Animals

The current in vitro study only used blood samples from animals listed in Table S1. These RMs, chronically infected with clade C SHIV, were part of our ongoing study to establish and optimize a model to test experimental vaccine approaches against the most frequent clade of HIV worldwide, and data from each of these animals have been published previously [19], [24]–[27]. Animal experiments were carried out in strict accordance with the recommendations in the Guide for the Care and Use of Laboratory Animals of the U.S. Public Health Services/National Institutes of Health, as well as according to the recommendations in the Weatherall report on ‘‘The Use of Non-human Primates in Research’’ (http://www.acmedsci.ac.uk/images/project/nhpdownl.pdf). The protocol was approved by the Committee on the Ethics of Animal Experiments of Emory University (IACUC ID: YER-2001401-111814; Emory University Animal Welfare Assurance Number A3180-01). The rhesus monkeys were housed at the Yerkes National Primate Research Center (YNPRC, Emory University, Atlanta, GA). The animals were housed indoors with a 12 hour light/dark cycle, in individual cages but in visual and auditory contact with other RMs, were fed monkey chow (Purina) ad libitum supplemented daily with fresh fruit. Standard enrichment was provided by the YNPRC enrichment staff including perches, rubber toys and varied treats such as peanuts and cereals. Blood was collected under ketamine or Telazol anesthesia from the femoral vein. YNPRC facilities are fully accredited by the Association for Assessment and Accreditation of Laboratory Animal Care International. Animal experiments were approved by the Institutional Animal Care and Use Committees at Emory and the Dana-Farber Cancer Institute via a Collaborating Institution Animal Use Agreement. Because the experiments described here involved a virus that may cause an incurable disease, such as AIDS, discomfort, stress and pain may occur. Animals were closely monitored and observed for development of disease at least twice daily. Animals determined to be under stress or in discomfort, were administered appropriate anesthetics and/or analgesics as directed by the clinical veterinary staff. If stress cannot be alleviated, the animals are euthanized.

### Collection of Human PBMC

This study made use of anonymous human blood donor samples for in vitro neutralization tests. These cells were derived from the blood bank at Brigham and Women’s Hospital (Boston, MA), where leukocytes were collected in Trima collars during platelet pheresis. The donors were prescreened and found to be negative for blood-borne pathogens, including HIV-1, hepatitis, and others. The use of Trima-collar derived PBMC for in vitro neutralization assays was reviewed by the IRB; this use of human PBMC was considered to be exempt.

### Envelope Proteins and Peptides

HIV envelope proteins of strains UG37, BaL, IIIB, CN54 and UG21 along with consensus clade C peptides were kindly provided by the NIH AIDS Research and Reference Reagent Program (ARRRP). SHIV-1157ip and SIVmne envelope proteins were generated by recombinant vaccinia virus technology [28].

### Differential Biopanning

#### Affinity purification of polyclonal Abs specific for native vs. denatured antigens

Affinity purification was performed in 96-well plates coated overnight with HIV gp160_SHIV-1157ip_ under native or denaturing conditions. On the next day, plates were washed, blocked and incubated overnight with plasma of monkey RKl-8. The following day, plates were extensively washed, and bound Abs were eluted by pH shift. Identical fractions were combined, concentrated and used for biopanning of random peptide phage libraries.

#### Biopanning

Biopanning of random peptide phage display libraries with polyclonal clade C SHIV-infected RM serum is described elsewhere [29]. Briefly, paramagnetic beads (Dynabeads M-280 tosyl-activated; Invitrogen) were coated with rabbit anti-monkey IgG (Sigma-Aldrich) and pre-incubated with serum Abs affinity-purified using native gp160_SHIV-1157ip_ (positive selection). After an overnight incubation with 10 µl of the original phage display peptide libraries (7mer, cyclic 7mer, 12mer; New England Biolabs), beads were intensively washed and bound phages were eluted by a 10 min pH shift using 0.2 M glycine-HCl pH 2.2 supplemented with 1 mg/ml BSA (Sigma-Aldrich) and subsequently neutralized with 1 M Tris-HCl pH 9.1 (Sigma-Aldrich). Eluted phages were used for negative selection with serum Abs that had been affinity-purified with denatured gp160_SHIV-1157ip_ (boiled for 3 min in carbonate buffer (pH 9.6) containing 0.05 M TCEP, *tris*(2-carboxyethyl)phosphine (Pierce) and 2% SDS (Sigma-Aldrich). Phages remaining in the supernatant after negative selection were amplified for 4.5 h in *E. coli* (ER2738, New England Biolabs), precipitated overnight at 4°C (20% PEG-8000/2.5 M NaCl; Fisher Scientific), collected, precipitated a second time and subjected to two more positive/one more negative selection rounds. Eluted phages from the third positive selection were titered on LB plates and single clones were picked and tested by phage ELISA for specific binding. Single-stranded DNA of positive clones was isolated and sequenced to deduce the sequences of the peptide inserts, which were grouped into motifs and assigned to sequences of the Env-C protein, where possible.

### Molecular Modeling and Structure Images

Protein modeling and energy calculations were performed using Discovery Studio (Accelrys Software, Inc.) based on a sequence alignment of the V3-mimotope with the core structure corresponding to the X-ray structure of ZAM18 V3 peptide in complex with Fab2557 [13] (pdb code 3MLU). Energies were calculated using CHARMM (Chemistry at Harvard Macromolecular Mechanics). We introduced solvent factors using the implicit of distance-dependent dielectrics model and performed energy minimization of the mimotope (Steepest Descent following by Conjugate Gradient).

Molecular graphics image for antibody [30] (pdb code 1IGT) was produced using the UCSF Chimera package from the Resource for Biocomputing, Visualization, and Informatics at the University of California, San Francisco (supported by NIH P41 RR001081). Model for mWasabi was generated by Swiss-Model Workspace [31] (Swiss Institute of Bioinformatics & the Biozentrum University of Basel) and the image was produced with UCSF Chimera package.

### Generation of pET-mWasabi.II-mime Vector and mWasabi-mimotope Fusion Expression (Figure S1)

First, sequences encoding mWasabi with a N-terminal His_6_-tag were amplified from the parental plasmid pNCS-mWasabi (Allele Biotechnology) with primers to introduce NdeI and EcoRI restriction sites (Table S5) and cloned into the pET-22b(+) vector (Novagen). The resulting plasmid was termed pET-mWasabi.II. Next, mimotopes plus flanking regions were amplified from phage DNA with primers to introduce EcoRI and HindIII restriction sites (Table S5) followed by cloning into pET-mWasabi.II at the C-terminus of the fluorescent protein. The mWasabi-mimotope fusion proteins were expressed in BL21 *E.coli* and purified on the Ni^2+^-agarose according to the manufacturer instruction.

### ELISAs

Phage binding ELISA and mimotope fusion protein ELISA were performed as published [29].

#### ELISAs with cell culture supernatants and purified mAbs

ELISA plates were coated with 50–100 ng/well of antigens (mimotope fusion proteins, HIV proteins or V3 loop consensus peptides) in carbonate buffer, pH 9.6. After washing, plates were blocked with 2% bovine serum albumin (BSA) (Sigma-Aldrich), 0.05% Tween-PBS (blocking buffer). Plates were then incubated with cell supernatants diluted with blocking buffer or purified mAbs diluted at 1 µg/ml with several consecutive 1∶4 dilutions in blocking buffer. After washing, the plates were developed by incubation for 1 h with goat HRP-conjugated anti-human IgG (Jackson ImmunoResearch) and by adding 100 µl of *o*-phenylenediamine or TMB solution. Optical densities were measured at the appropriate wavelength using an ELISA microplate reader (Mithras LB 940, Berthold Technologies).

#### “Denatured” ELISA

Wells of 96-well plates were coated with gp160_SHIV-1157ip_ under native or denaturing conditions. For denaturation, antigen was diluted to at 0.5 µg/ml into carbonate buffer (pH 9.6) containing 0.05 M TCEP and 2% SDS and boiled for 3 min. Antigen in coating buffer served as “native”. Subsequent ELISAs were performed similarly as described above.

#### Competitive ELISA

The interaction of anti-V3 loop mAbs with solid-phase HIV_CN54_ gp120 was inhibited by peptides corresponding to the V3 loop of consensus clade C gp120 sequence (Figure S4A). A negative peptide control consisted of a scrambled C-terminal gp120 peptide (24 amino acids long; synthesized by the Molecular Biology Core Facilities, DFCI).

### Primer Design

To design primers specific for RM Ig genes (Table S3), we analyzed RM Ig V gene sequences obtained in our group as well as sequences available through public databases, IMGT, the international ImMunoGeneTics information system® (IMGT/LIGM-DB) and NCBI, The National Center for Biotechnology Information. Sequence analysis and germline gene identification were performed using the IMGT-V-Quest software; sequence alignment was done with DNAStar Lasergene software package (DNAStar Inc). In addition, using known sequences of rare human V genes, the RM genome was blasted to identify potential counterparts. To validate newly designed primers, V_H_ and V_L_ genes were amplified using cDNA derived from mRNA isolated from the mixture of bone marrow B cells of 10 RMs. The resulting PCR products were cloned into the sequencing plasmid vector, pCR4-TOPO (Invitrogen), and sequenced. Sequences of 300 randomly chosen clones were analyzed.

### Mimotope-specific Memory B-cell Isolation

RM PBMC were obtained from YNPRC by overnight shipping in BD Vacutainer® CPT™ Cell Preparation Tubes (Beckton Dickinson, BD). Upon arrival, cells were washed with RPMI 1640 (Invitrogen) supplemented with 15% fetal calf serum (FCS) (Sigma-Aldrich) followed by PBS containing 0.2% BSA (Sigma-Aldrich). Then, cells were incubated with APC-Cy™7 mouse anti-human CD3 (BD Pharmingen), APC mouse anti-human CD27 (BD Pharmingen), PE-Cy™7 mouse anti-human IgG (BD Pharmingen), anti-CD19-R-Phycoerythrin (Beckman Coulter) and mWasabi-mimotope fusion protein for 30 min on ice in PBS-BSA. Controls were performed with isotype controls and mWasabi. Cells were then washed twice with ice cold PBS-BSA and single-cell sorted (FACSAria II (BD Biosciences)) into 96-well PCR plates as described [32]. Live single-cell sorting of RM mimotope-specific memory B cells under appropriate biocontainment was available through the Harvard CFAR Core facility. Plates were snap frozen on dry ice immediately after the completion of sorting.

### Single B-cell RT-PCR and Expression Vector Cloning

cDNA synthesis and Ig amplification were performed as previously described [32], with following modifications. The frozen plates with single memory B cells were thawed, and reverse transcription was performed by adding 3 µl of random hexamer primers (Applied Biosystems) at 50 µM, 1 µl of 10 mM dNTP mix (Invitrogen), 0.0625 µl of Igepal CA-630 (Sigma), 40 units of RNaseOUT™ (Invitrogen), 1.25 µl of 0.1 DTT (Invitrogen) and 0.25 µl of SuperScript III (Invitrogen) into each well. Reaction conditions for reverse transcription were as follows: 42°C for 10 min, 25°C for 10 min, 50°C for 60 min and 94°C for 5 min. The cDNA plates were stored at −20°C until further use. The IgH, Igλ and Igκ V genes were amplified independently by semi-nested PCR starting from 3 µl of cDNA as a template. All PCRs were performed in 96-well PCR plates in a total volume of 50 µl containing water, 5 µl of 10× buffer, 1 µl of dNTP mix, each at 10 mM, 1 µl of MgCl_2_ at 25 mM (Qiagen), by 1 µl of forward primer mix and reverse primer (Table S3) at 10 µM, and 0.4 µl of HotStart Taq DNA polymerase (Qiagen). The PCR thermocycler program was: 95°C for 15 min; 50 cycles (95°C for 30 sec, 50°C for Igκ and Igλ or 54°C for IgH for 30 sec and 72°C for 1 min); 72°C for 10 min. For PCR2, 3 µl of PCR1 product were used as a template. Forward primers were as in PCR1 and reverse primer specific for PCR2 (Table S3). Fifty cycles were used with the same parameters as those for the first round, with annealing temperature of 52°C for Igκ and Igλ and 57°C for IgH for 30 sec. After PCR2, the fragments for matching heavy/light chain pairs were isolated and subjected to direct sequencing with the reverse PCR2 primer. PCR products that represented productive IgH, Igλ or Igκ rearranged sequences were reamplified from PCR1 using “cloning” primers. These primers contained unique restriction digest sites, a Kozak motif, leader peptide coding region and sequences complementary to FR1. After amplification, DNA fragments were gel purified, digested and cloned into pFUSE2-CLIg-hk, pFUSE2-CLIg-hl2 and pFUSE2-CHIg-hG1 (all from Invivogen) containing the backbones of human Igγ1, Igκ and Igλ, respectively, thus giving rise to chimeric simian-human mAbs.

### Ab Production and Purification

Full-length IgG1 mAbs were produced by transient cotransfection of the paired heavy and light chain pFUSE plasmids into 293-F cells (Invitrogen) grown in serum-free FreeStyle™ 293 Expression Medium (Gibco® Invitrogen) using the *Trans*IT-PRO™ Transfection Kit (Mirus Bio). Cells were cultivated for 3 days at 37°C/8% CO_2_ with continuous shaking at 135 rpm. Supernatants were collected, filtered through 0.22 µm filters and supplemented with Halt Protease Inhibitor Cocktail (Thermo Fisher) and 100× penicillin-streptomycin solution (Gibco® Invitrogen). Next, supernatants were tested for binding to HIV Env and mimotopes, and positive mAbs were affinity-purified using Protein A agarose (GE Healthcare) according to manufacturer’s instructions. IgG concentrations were determined by measuring absorbance at 280 nm on Nanodrop 1000 (Thermo Scientific) using the IgG default protocol.

### Virion Binding Assay

ELISA plates (Nunc) were coated with 5 µg/ml of goat anti-human IgG Fc specific Ab (Jackson ImmunoResearch) overnight at 4°C. After blocking and washing, mAbs were added at 5 µg/ml and incubated for 2 h. The plates were washed, SHIV-1157ipEL-p was added to the mAbs and incubated for 20 h, after which the plates were washed again and incubated with 0.5% Triton X-100 for 1 h to release p27 from the virus bound to the various mAbs. The amount of p27 released was determined using a p27 SIV capture kit (ABL, Inc).

### Neutralization Assays

The TZM-bl and M7-Luc assays were performed as described elsewhere [33] and the human PBMC assay was performed as reported [34].

#### A3R5 cell assay

A3R5 (A3.01/R5.6) cells are derived from the CEM human lymphoblastoid cell line A3.01 [35] and engineered to express CCR5 (McLinden RJ, Chenine AL, LaBranche C, Perfetto S, Ochsenbauer C, Kappes J, Montefiori, DC, Kim JH. “Novel CD4+/CCR5+/CXCR4+ human T-cell line shows enhanced sensitivity of HIV-1 to neutralization by sCD4, mAbs and HIV-1-positive sera”, manuscript in preparation); cells were obtained from Drs. Jerome Kim and Robert McLinden at the US Medical HIV Research Program. The A3R5 assay was performed with Env.IMC.LucR viruses [36]. Neutralization titers reflect the sample concentration at which relative luminescence units (RLU) were reduced by 50% compared to RLU in virus control wells after subtraction of background activity in cell control wells.

### Reactivity with Autoantigens

Autoreactivity was tested with an anti-dsDNA EIA kit, anti-Sm/RNP EIA kit, anti-Sm EIA kit, autoimmune EIA anti-SS-A/Ro Test, autoimmune EIA anti-SS-B/La test, Bio-Rad Kallestad ANA screen (all Bio-Rad) and QUANTA Lite® ACA IgG III (INOVA Diagnostics). Assays were performed on automated PhD System (Bio-Rad) and DSX™ System (Dynex Technologies).

## Supporting Information

Figure S1 **Cloning of mimotopes into the mWasabi backbone.** mWasabi and mimotope fragments were amplified using specific primers to introduce appropriate restriction sites to insert both sequences (boxed) into the pET-22b(+) vector. mWasabi was cloned first and the resulting vector pET-mWasabi.II served as an acceptor of different mimotopes. After transformation of *E.coli* with the resulting plasmids, mWasabi-mimotope fusion proteins were expressed under IPTG induction and purified from bacterial cell lysates by metal-affinity chromatography. pNCS-mWasabi was obtained from Allele Biotech.
(TIF)

Figure S2 **Binding analysis of 12 293F cell supernatants to mimotope fusion proteins, mWasabi-Tc.2 and HIV Env.** Supernatants of 293F cells transfected with plasmids encoding cognate heavy and light immunoglobulin chains were collected 72 h post-transfection, diluted with blocking buffer (Material and Methods) and incubated with mWasabi-Tc.2, mWasabi or HIV_CN54_ gp120. The anti-V3 loop mAb HGN194 [16] and monkey RJa-9 serum were used as positive controls. Supernatant from non-transfected 293F cells (0 sup.) served as negative control. Each data point represents the mean ± s.e.m. (n  = 3).
(TIF)

Figure S3 **Binding of mAbs 33B2 and 33C6 to HIV Env of different clades.** Plates were coated with envelope proteins and probed with different dilutions of mAbs 33B2 and 33C6. HIV Env proteins were derived from the following strains: clade A, UG37; B, BaL and IIIB; C, CN54 and 1157ip; D, UG21. SIVmne gp160 was used as negative control. MAb HGN194 served as positive and Fm-6 [37] as negative isotype controls, respectively. Each data point represents the mean ± s.e.m. (n  = 3).
(TIF)

Figure S4 **Inhibition of binding of mAbs to HIV_CN54_ gp120 by consensus clade C peptides representing the V3 loop region.** ELISA plates were coated with gp120 and exposed to mAbs mixed with V3 loop peptides (9258, 9259, 9260, 9261, 9262, and 9263) or control peptide representing the scrambled C-terminus of HIV gp120. Each data point represents the mean ± s.e.m. (n  = 3). (A) Amino acid sequences of linear consensus clade C peptide representing the V3 loop of gp120; (B) inhibition of binding of mAb 33B2; and (C) inhibition of binding of mAb 33C6.
(TIFF)

Figure S5 **Alignment of 33B2 and 33C6 VH with human (HU-IGHV5-51) and rhesus monkey (RM-IGHV-5-51) germline amino acid sequences and calculation of mutation frequency versus rhesus monkey germline.** Red amino acids, divergence from the rhesus monkey germline; green, divergence from the human germline.
(DOC)

Figure S6 **Alignment of 33B2 and 33C6 VL with human (HU-IGLV1-50 and HU-IGLV1-47) and rhesus monkey (RM-IGLV1-50 and RM-IGLV1-47) germline amino acid sequences and calculation of mutation frequency versus rhesus monkey germline.** Red amino acids, divergence from the rhesus monkey germline; green, divergence from the human germline.
(DOC)

Table S1 **Treatment history and clinical parameters for cohort of RMs used for the study.**
(DOC)

Table S2 **IC_50_ neutralization titers of RM sera.**
(DOC)

Table S3 **Primers for amplification of rhesus monkey immunoglobulin V heavy and light chain genes.**
(DOC)

Table S4 **Frequency of RM V gene usage.**
(DOC)

Table S5 **Primers for amplification of mWasabi and mimotopes.**
(DOC)
