# Supplementary figures and images for: Isolation of Monoclonal Antibodies with Predetermined Conformational Epitope Specificity
Source: PLoS One. 2012 Jun 21;7(6):e38943. doi: 10.1371/journal.pone.0038943 (PMC3380854; doi:10.1371/journal.pone.0038943)

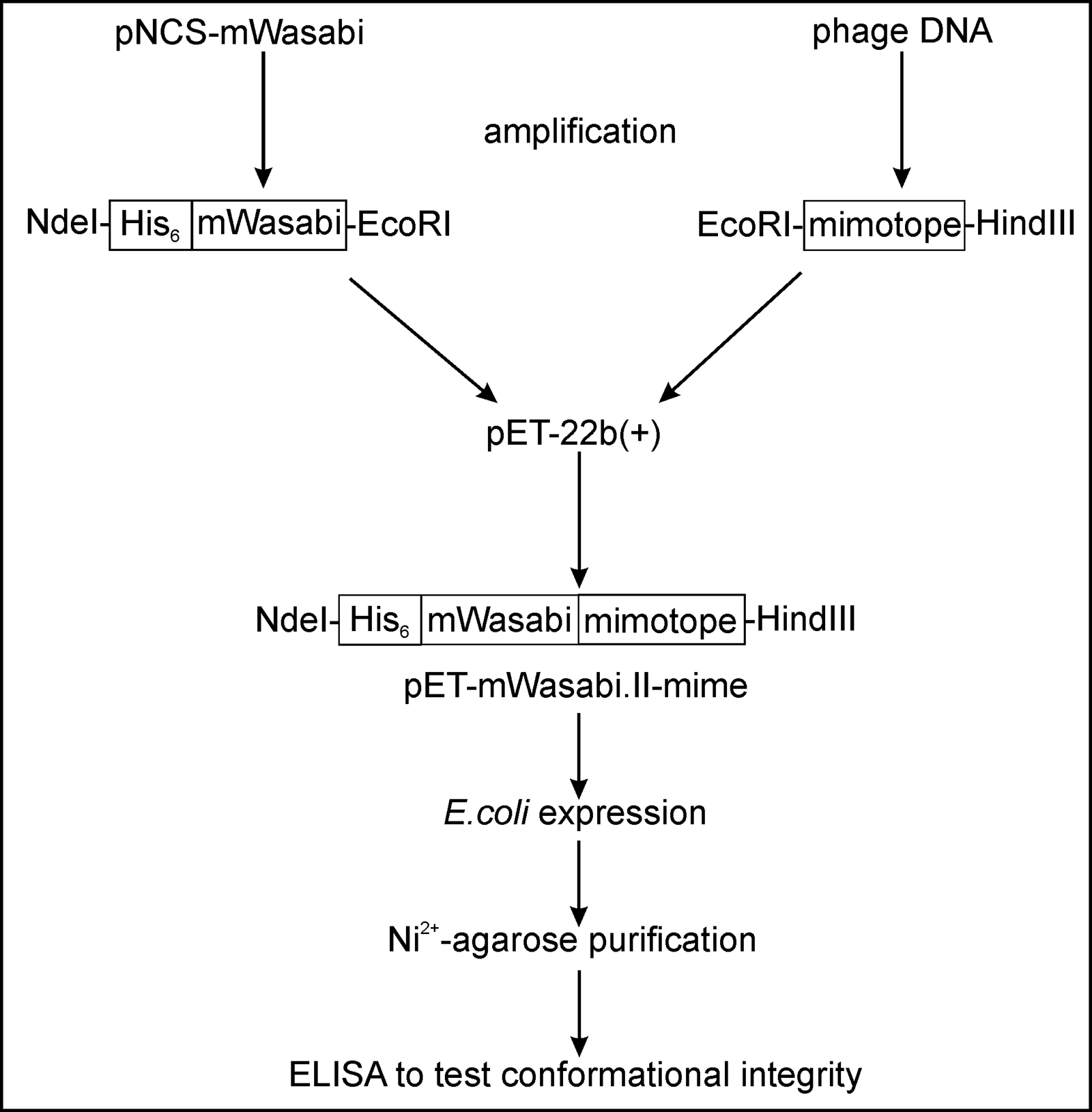

Supplement: Figure S1 — Cloning of mimotopes into the mWasabi backbone. mWasabi and mimotope fragments were amplified using specific primers to introduce appropriate restriction sites to insert both sequences (boxed) into the pET-22b(+) vector. mWasabi was cloned first and the resulting vector pET-mWasabi.II served as an acceptor of different mimotopes. After transformation of E.coli with the resulting plasmids, mWasabi-mimotope fusion proteins were expressed under IPTG induction and purified from bacterial cell lysates by metal-affinity chromatography. pNCS-mWasabi was obtained from Allele Biotech. (TIF) [file pone.0038943.s001.tif]

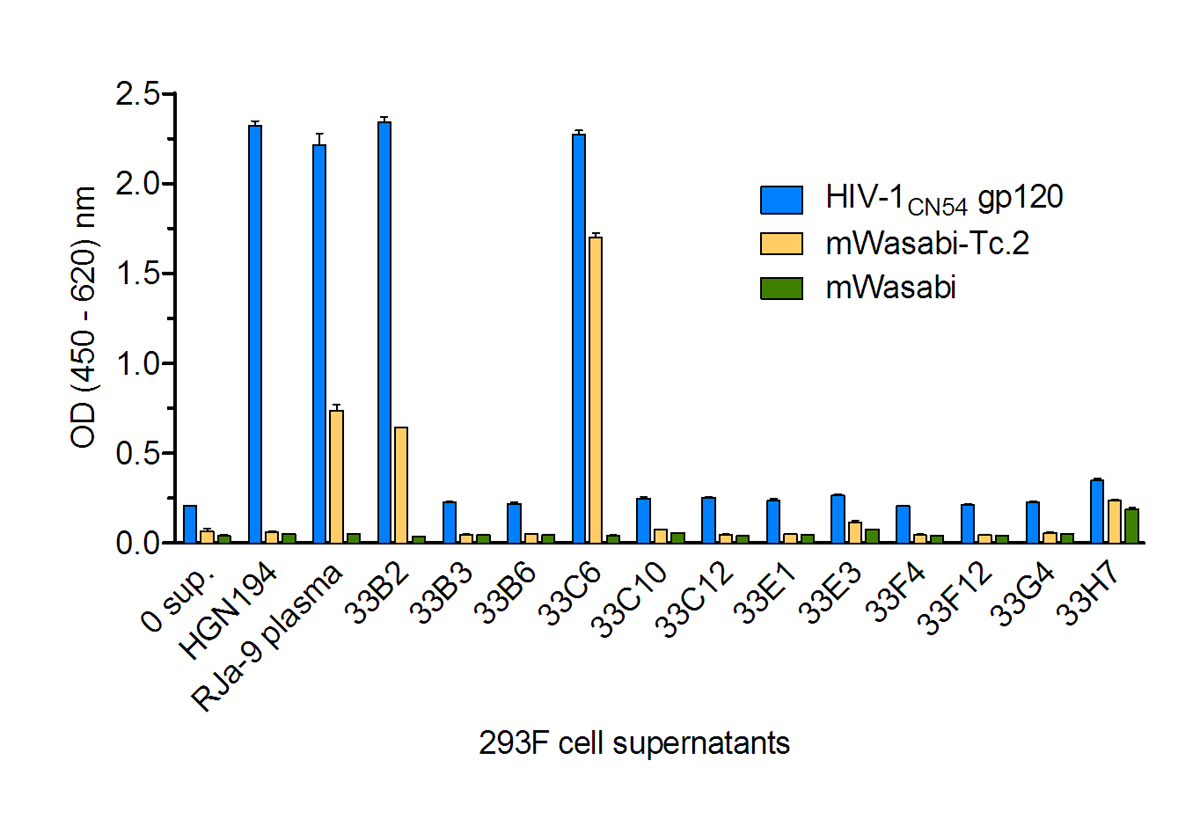

Supplement: Figure S2 — Binding analysis of 12 293F cell supernatants to mimotope fusion proteins, mWasabi-Tc.2 and HIV Env. Supernatants of 293F cells transfected with plasmids encoding cognate heavy and light immunoglobulin chains were collected 72 h post-transfection, diluted with blocking buffer (Material and Methods) and incubated with mWasabi-Tc.2, mWasabi or HIVCN54 gp120. The anti-V3 loop mAb HGN194 [16] and monkey RJa-9 serum were used as positive controls. Supernatant from non-transfected 293F cells (0 sup.) served as negative control. Each data point represents the mean ± s.e.m. (n = 3). (TIF) [file pone.0038943.s002.tif]

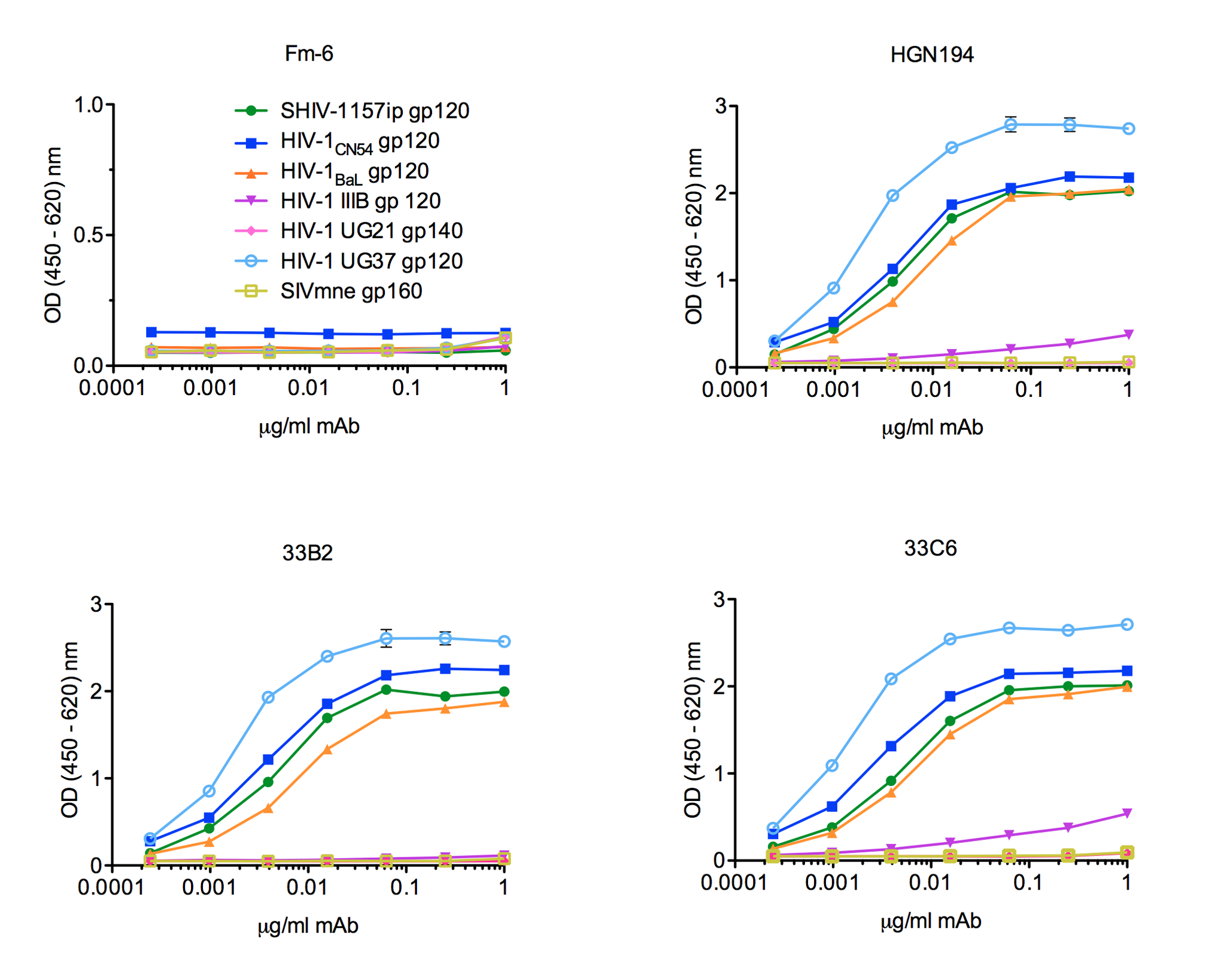

Supplement: Figure S3 — Binding of mAbs 33B2 and 33C6 to HIV Env of different clades. Plates were coated with envelope proteins and probed with different dilutions of mAbs 33B2 and 33C6. HIV Env proteins were derived from the following strains: clade A, UG37; B, BaL and IIIB; C, CN54 and 1157ip; D, UG21. SIVmne gp160 was used as negative control. MAb HGN194 served as positive and Fm-6 [37] as negative isotype controls, respectively. Each data point represents the mean ± s.e.m. (n = 3). (TIF) [file pone.0038943.s003.tif]

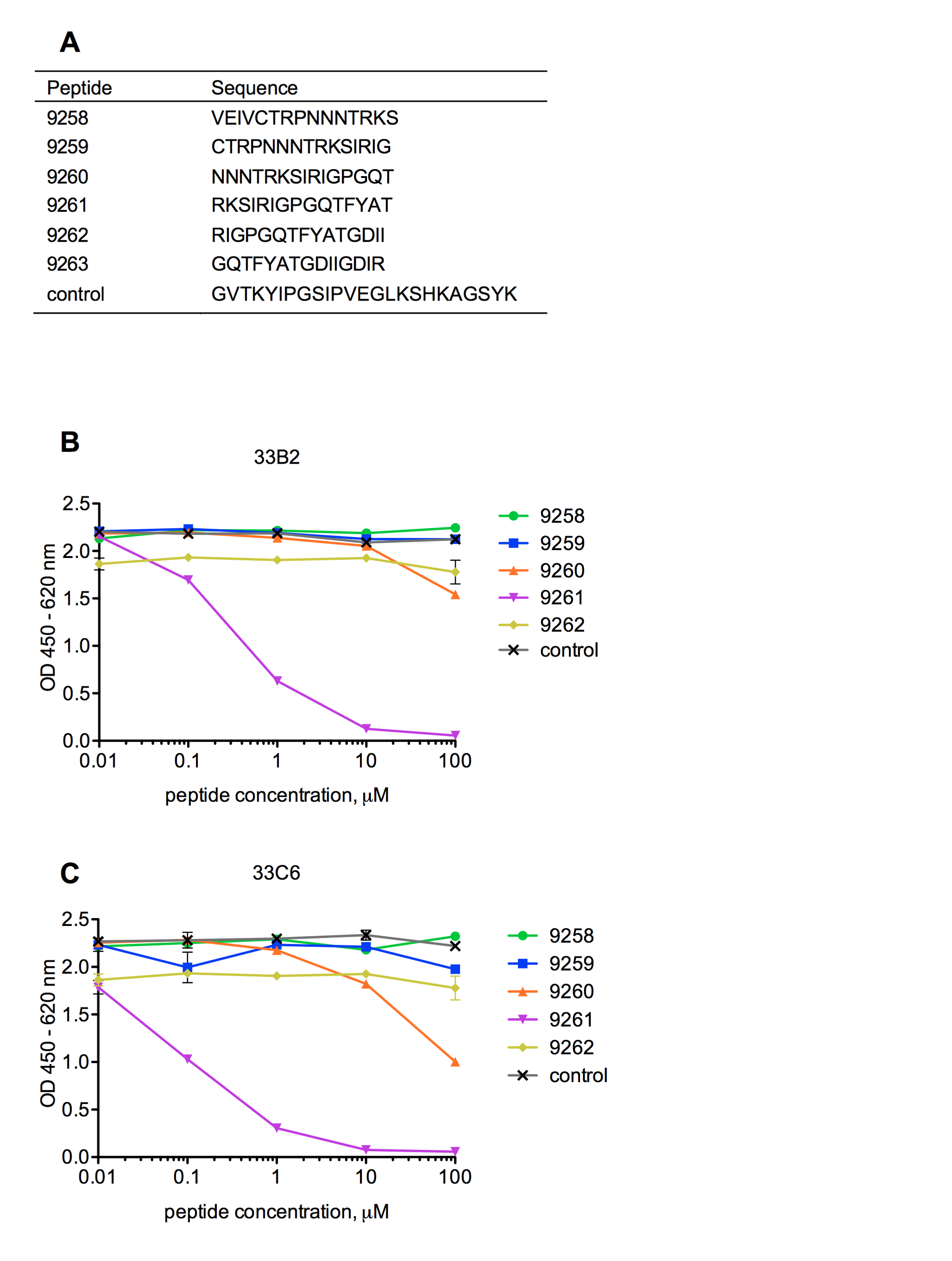

Supplement: Figure S4 — Inhibition of binding of mAbs to HIVCN54 gp120 by consensus clade C peptides representing the V3 loop region. ELISA plates were coated with gp120 and exposed to mAbs mixed with V3 loop peptides (9258, 9259, 9260, 9261, 9262, and 9263) or control peptide representing the scrambled C-terminus of HIV gp120. Each data point represents the mean ± s.e.m. (n = 3). (A) Amino acid sequences of linear consensus clade C peptide representing the V3 loop of gp120; (B) inhibition of binding of mAb 33B2; and (C) inhibition of binding of mAb 33C6. (TIFF) [file pone.0038943.s004.tiff]
