# Supplementary material for: Isolation of Monoclonal Antibodies with Predetermined Conformational Epitope Specificity
Source: PLoS One. 2012 Jun 21;7(6):e38943. doi: 10.1371/journal.pone.0038943 (PMC3380854; doi:10.1371/journal.pone.0038943)
Supplement: Figure S5 — Alignment of 33B2 and 33C6 VH with human (HU-IGHV5-51) and rhesus monkey (RM-IGHV-5-51) germline amino acid sequences and calculation of mutation frequency versus rhesus monkey germline. Red amino acids, divergence from the rhesus monkey germline; green, divergence from the human germline. (DOC) [file pone.0038943.s005.doc]

10 20 30 40 50 60 70 80

---------+---------+---------+---------+---------+---------+---------+---------+

HU-IGHV5-51 EVQLVQSGAEVKKPGESLKISCKGSGYSFTSYWIGWVRQMPGKGLEWMGIIYPGDSDTRYSPSFQGQVTISADKSISTAY

RM-IGHV5-51 EVQLVQSGAEVKRPGESLKISCKTSGYSFTSYWISWVRQMPGKGLEWMGAIDPSDSDTRYSPSFQGQVTISADKSISTAY

33B2-VH EVQLVQSGVEVKRPGDSLRIPCKTSGDTITSLWINWVRQMPGKGLEWMGMIYPEDSDVTFSPSFQGQVTMSVDKSINTAY

33C6-VH QVQLVQSGPEVKRPGDSLTIPCKTSGFSSNYLWINWVRQMPGKGLEWMGMIYLGDSDTTYSPSFRGQVTMSVDKSIKTAY

90

---------+-------

HU-IGHV5-51 LQWSSLKASDTAMYYCA 97

RM-IGHV5-51 LQWSSLKASDTATYYCA 97

33B2-VH LRWNSLKASDTATYYCA 97

33C6-VH LQWGNLKASDTATYYCA 97

| Ab | Mutation frequency  vs. RM germline, % |
| --- | --- |
| 33B2 VH | 14.6 (20/97) |
| 33C6 VH | 17.6 (22/97) |
