# Supplementary material for: Isolation of Monoclonal Antibodies with Predetermined Conformational Epitope Specificity
Source: PLoS One. 2012 Jun 21;7(6):e38943. doi: 10.1371/journal.pone.0038943 (PMC3380854; doi:10.1371/journal.pone.0038943)
Supplement: Figure S6 — Alignment of 33B2 and 33C6 VL with human (HU-IGLV1-50 and HU-IGLV1-47) and rhesus monkey (RM-IGLV1-50 and RM-IGLV1-47) germline amino acid sequences and calculation of mutation frequency versus rhesus monkey germline. Red amino acids, divergence from the rhesus monkey germline; green, divergence from the human germline. (DOC) [file pone.0038943.s006.doc]

10 20 30 40 50 60 70 80

---------+---------+---------+---------+---------+---------+---------+---------+---------

HU-IGLV1-50 QSVLTQPPSVSGAPGQRVTISCTGSSSNIGAGYVVHWYQQLPGTAPKLLIYGNSNRPSGVPDQFSGSKSGTSASLAITGLQSEDEADYY 89

RM-IGLV1-50 QSVLTQPPSVSGDPGQRVTISCTGSSSNIG--YDVYWYQQLPGTAPKLLIYENNKRPSGVSDRFSGSKSGTSASLTITGLQSEDEAEYY 88

33B2-VL QSVLTQPPSVSGAPGQTVTISCTGSSSNIGADRYVSWYQQFPGTAPKLLIFENNKRPSGISDRFSGSKSGSSASLTITGLQSEDEADYY 89

10 20 30 40 50 60 70 80

---------+---------+---------+---------+---------+---------+---------+---------+---------

HU-IGLV1-47 QSVLTQPPSASGTPGQRVTISCSGSSSNIG-SNYVYWYQQLPGTAPKLLIYRNNQRPSGVPDRFSGSKSGTSASLAISGLRSEDEADYY 79

RM-IGLV1-47 QSVLTQPPSVSGDPGQRVTISCTGSSSNIG-GYDVYWYQQLPGTAPKLLIYENNKRPSGVSDRFSGSKSGTSASLTITGLQSEDEAEYY 79

33C6-VL SYVLTQPPSASGAPGQRVTISCTGGSSNIGANRYVSWYQQLPGKAPKLLIFEDDKRHSGVSGRFSGSKSGSSASLTITGLRSEDEADYY 80

| Ab | Mutation frequency  vs. RM germline, % |
| --- | --- |
| 33B2 VL | 9.7 (11/89) |
| 33C6 VL | 15.2 (18/89) |
