# Supplementary material for: Isolation of Monoclonal Antibodies with Predetermined Conformational Epitope Specificity
Source: PLoS One. 2012 Jun 21;7(6):e38943. doi: 10.1371/journal.pone.0038943 (PMC3380854; doi:10.1371/journal.pone.0038943)
Supplement: Table S1 — Treatment history and clinical parameters for cohort of RMs used for the study. (DOC) [file pone.0038943.s007.doc]

**Table S1. Treatment history and clinical parameters for cohort of RMs used for the study**

| Monkey | Virus exposure | Other treatments | Viral RNA copies/ml | | Current CD4 count | Number months infected | Current CD4/CD8 ratio | Clinical status | Ref. |
| --- | --- | --- | --- | --- | --- | --- | --- | --- | --- |
|  |  |  | Peak | Current |  |  |  |  |  |
| RKl-8† | SHIV-1157i  passage | - | 1.9 x 10^7^ | - | - | 73 | - | systemic infection, death from AIDS | 24 |
| RAo-8 | SHIV-1157i  passage | - | 2.8 x 10^7^ | <50 | 789 | 122 | 2.13 | systemic infection, LTNP | 24 |
| RJa-9 | SHIV-1157ip  oral titration | *S. mansoni* | 3.7 x 10^6^ | <50 | 476 | 114 | 1.13 | systemic infection, LTNP | 25 |
| RMf-9 | SHIV-1157ip  oral titration | *S. mansoni* | 7.2 x 10^7^ | <50 | 467 | 114 | 0.93 | systemic infection, LTNP | 25 |
| RLu-9 | SHIV-1157ip  SHIV-1157ipd3N4 | HIV1084i gp160 (2x)  SIV Gag-Pol particles (2x)  HIV Tat (2x) | 7.8 x 10^5^ | <50 | 415 | 71 | 0.97 | systemic infection, LTNP | 26 |
| RHo-10 | SHIV-1157ipd3N4 | - | 7.1 x 10^7^ | 700 | 242 | 72 | 0.81 | persistently viremic | 27 |
| REk-11 | SHIV-1157ipEL-p | - | 4.4 x 10^5^ | 24,650 | 324 | 40 | 0.45 | persistently viremic | 19 |
| RIj-11 | SHIV-1157ipEL-p | - | 1.8 x 10^5^ | 300 | 510 | 40 | 1.04 | persistently viremic | 19 |
| RKa-11 | SHIV-1157ipEL-p | - | 2.4 x 10^6^ | 1,550 | 757 | 33 | 1.08 | persistently viremic | 19 |

† - died of AIDS
