# Supplementary material for: Isolation of Monoclonal Antibodies with Predetermined Conformational Epitope Specificity
Source: PLoS One. 2012 Jun 21;7(6):e38943. doi: 10.1371/journal.pone.0038943 (PMC3380854; doi:10.1371/journal.pone.0038943)
Supplement: Table S2 — IC50 neutralization titers of RM sera. (DOC) [file pone.0038943.s008.doc]

**Table S2. IC_50_ neutralization titers of RM sera**

|  | IC_50_, μg/ml (TZM-bl cells) | | | IC_50_, μg/ml (hPBMC) | |
| --- | --- | --- | --- | --- | --- |
| Monkey | SHIV SF162.P4 (tier 1) | SHIV-1157ipEL-p (tier 1) | MW965.26 (tier 1) | SHIV-2873Nip (tier 2) | SHIV-1157ipd3N4 (tier 2) |
| RAo-8 | 976 | 2,269 | 43,740 | >640 | >640 |
| REk-11 | >43,740 | 14,151 | >43,740 | ND | ND |
| RHo-10 | 13,824 | 886 | 11,491 | >640 | 532 |
| RIj-11 | 1,284 | 1,544 | 20,614 | ND | ND |
| RJa-9 | 2,015 | 738 | 17,301 | 2,048 | >10,240 |
| RKa-11 | 14,549 | 4,362 | >43,740 | ND | ND |
| RLu-9 | 1,668 | 461 | 4,349 | >640 | >640 |
| RMf-9 | 931 | 464 | 19,025 | >640 | >640 |

Neutralization assay for tier 1 viruses was performed in TZM-bl cell assay and for tier 2 viruses in human PBMC. ND, not determined.
