# Supplementary material for: Isolation of Monoclonal Antibodies with Predetermined Conformational Epitope Specificity
Source: PLoS One. 2012 Jun 21;7(6):e38943. doi: 10.1371/journal.pone.0038943 (PMC3380854; doi:10.1371/journal.pone.0038943)
Supplement: Table S3 — Primers for amplification of rhesus monkey immunoglobulin V heavy and light chain genes. (DOC) [file pone.0038943.s009.doc]

**Table S3. Primers for amplification of rhesus monkey immunoglobulin V heavy and light chain genes**

| Forward primer | 5’ – 3’ sequence |
| --- | --- |
| VH-1 | SAGGWSCAGCTGGTRCAATCCGG |
| VH-2 | CAGGTGACCTTGAAGGAGTCTGG |
| VH-3/5/7 | SAGGTGCAGYTGGTGSAGTCTGG |
| VH-4/6 | CAGGTGCARCTGCAGGAGTCRGG |
| VH-5 | GAGGTGCAGCTGGTGCAGTCTGG |
| VH-6 | CAGGTACAGCTGCAGCAGTCAGG |
| VH-7 | CAGGTGCAGCTGGTGCAATCTGG |
| Vλ-1 | CAGTCTGTRCTGACVCAGCCDCC |
| Vλ-2 | CAGKCTGCCCYGAYTCAGYCTCC |
| Vλ-3A | TCCTCTGGGCTGACTCAG |
| Vλ-3B | TCCTMTGAGCTGACACAGCCDCC |
| Vλ-4 | CAGCYTGTGCTGACTCARTCGCC |
| Vλ-5 | MAGSCTRTGCTGACTCAGCCRRC |
| Vλ-6 | AATTTTATGCTGACTCAGCCC |
| Vλ-8/7 | CAGACTGTGGTGACYCAGGAGYC |
| Vλ-9 | CAGCYTGTGCTGACTCARCCACC |
| Vλ-10 | CAGGCAGGGCTGACTCAGCCACC |
| Vκ-1 | GACATYCAGATGWCCCAGTCTCC |
| Vκ-2 | GATAYTGTGATGACCCAGACTCC |
| Vκ-3 | SAAATWGTRWTGACKCAGTCTCC |
| Vκ-4 | GACATYGTGMTGACCCAGTCTCC |
| Vκ-5 | GAAACGACACTCACGCAGTCTCC |
| Vκ-6 | GAWRTTGTGMTGACWCAGTCTCC |
| Vκ-7 | GACATTGTGCTGACCCAGTCTCC |

| Reverse primer | 5’ – 3’ sequence |
| --- | --- |
| γ-PCR1 | GGACAGCCKGGAAGGTGTGC |
| γ-PCR2 | GCCTGAGTTCCACGACACGGTCAC |
| λ-PCR1 | CCGCGTACTTGTTGTTGCTCTGT |
| λ-PCR2 | CAGAGGAGGGCGGGAASAGA |
| κ-PCR1 | GAGGCAGTTCCAGATTTCAA |
| κ-PCR2 | GGTGCAGCCACAGCTCGTTTGAT |

N = A+G+C+T; V = A+C+G; D = A+T+G; B = T+C+G; H = A+T+C; W = A+T; S = C+G;

K = T+G; M = A+C; Y = C+T; R = A+G;
