# Supplementary material for: Isolation of Monoclonal Antibodies with Predetermined Conformational Epitope Specificity
Source: PLoS One. 2012 Jun 21;7(6):e38943. doi: 10.1371/journal.pone.0038943 (PMC3380854; doi:10.1371/journal.pone.0038943)
Supplement: Table S4 — Frequency of RM V gene usage. (DOC) [file pone.0038943.s010.doc]

**Table S4. Frequency of RM V gene usage.**

|  | Heavy, % | Kappa, % | Lambda, % |
| --- | --- | --- | --- |
| V1 | 27 | 40 | 10 |
| V2 | 18 | 18 | 13 |
| V3 | 14 | 12 | 41 |
| V4 | 25 | 13 | 6 |
| V5 | 5 | 14 | 16 |
| V6 | 7 | - | - |
| V7 | 4 | 3 | 6 |
| V8 | N.A. | N.A. | 4 |
| V9 | N.A. | N.A. | 2 |
| V10 | N.A. | N.A. | 1 |
| V11 | N.A. | N.A. | 1 |

*N.A., non-applicable.
