# Supplementary material for: Isolation of Monoclonal Antibodies with Predetermined Conformational Epitope Specificity
Source: PLoS One. 2012 Jun 21;7(6):e38943. doi: 10.1371/journal.pone.0038943 (PMC3380854; doi:10.1371/journal.pone.0038943)
Supplement: Table S5 — Primers for amplification of mWasabi and mimotopes. (DOC) [file pone.0038943.s011.doc]

**Table S5.** **Primers for amplification of mWasabi and mimotopes**

| Primer | 5’ – 3’ sequence |
| --- | --- |
| Fw-Wasabi | CATCGGCATATGCGCGGTTCTCATCAT |
| Rev-Wasabi | CGCCCGGAATTCCTTGTACAGCTCGTC |
| Fw-mimotope | CGTGAATTCGGCCCTTTAGTGGTACCTTTCTATTCTCACTCT |
| Rev-mimotope | GATAAGCTTCTAACTTTCAACAGTTTCCGCCGAACCTCC |

*Restriction sites are underlined.
